# Accuracy of the Hospital Anxiety and Depression Scale for Identifying Depression in Chronic Obstructive Pulmonary Disease Patients

**DOI:** 10.1155/2014/973858

**Published:** 2014-12-04

**Authors:** Christoph Nowak, Noriane A. Sievi, Christian F. Clarenbach, Esther Irene Schwarz, Christian Schlatzer, Thomas Brack, Martin Brutsche, Martin Frey, Sarosh Irani, Jörg D. Leuppi, Jochen Rüdiger, Robert Thurnheer, Malcolm Kohler

**Affiliations:** ^1^Division of Pulmonology, University Hospital of Zurich, 8091 Zurich, Switzerland; ^2^Medical Sciences Department, Uppsala University, 75237 Uppsala, Sweden; ^3^Division of Pulmonology, Cantonal Hospital of Glarus, 8750 Glarus, Switzerland; ^4^Division of Pulmonology, Cantonal Hospital of St. Gallen, 9007 St. Gallen, Switzerland; ^5^Division of Pulmonology, Clinical Barmelweid, 5017 Barmelweid, Switzerland; ^6^Division of Pulmonology, Cantonal Hospital of Aarau, 5000 Aarau, Switzerland; ^7^University Clinic of Internal Medicine, Cantonal Hospital Baselland and University of Basel, 4031 Basel, Switzerland; ^8^Division of Pulmonology, Cantonal Hospital of Münsterlingen, 8596 Münsterlingen, Switzerland

## Abstract

Psychological morbidity is common in chronic respiratory diseases. The diagnostic accuracy of the Hospital Anxiety and Depression Scale (HADS) and risk factors for comorbid depression in chronic obstructive pulmonary disease (COPD) are addressed. Consecutive COPD patients (GOLD stage I–IV, 40–75 years old) were enrolled in a multicentre, cross-sectional cohort study. Diagnosis of depression was ascertained through clinical records. Lung function, HADS score, 6-minute walking test (6-MWT), MRC dyspnoea score, and COPD Assessment Test (CAT) were evaluated. Two hundred fifty-nine COPD patients (mean age 62.5 years; 32% female; mean FEV1 48% predicted) were included. Patients diagnosed with depression (29/259; 11.2%) had significantly higher HADS-D and HADS-Total scores than nondepressed patients (median (quartiles) HADS-D 6 [4; 9] versus 4 [2; 7], median HADS-Total 14 [10; 20] versus 8 [5; 14]). Receiver-operating characteristic plots showed moderate accuracy for HADS-D, AUC 0.662 (95%CI 0.601–0.719), and HADS-Total, AUC 0.681 (95%CI 0.620–0.737), with optimal cut-off scores of >5 and >9, respectively. Sensitivity and specificity were 62.1% and 62.6% for HADS-D compared to 75.9% and 55.2% for HADS-Total. Age, comorbidities, sex, and lower airflow limitation predicted depression. The HADS exhibits low diagnostic accuracy for depression in COPD patients. Younger men with comorbidities are at increased risk for depression.

## 1. Introduction

Depression is a common comorbidity in chronic obstructive pulmonary disease (COPD) patients [1]. Impaired lung function is a risk factor for depression with up to 4 in 10 respiratory patients affected [2]. Mood and anxiety disorders in patients with COPD are likely underdiagnosed [3], emphasising the need for a reliable and accurate instrument in the recognition of depression. The Hospital Anxiety and Depression Scale (HADS [4]) was originally designed by Zigmond and Snaith in 1983 as a short, easy-to-use, 14-item screening tool for depression and anxiety symptoms in the hospital outpatient setting [5]. It is composed of two 7-item subscales (HADS-D and HADS-A for depression and anxiety, resp.) both ranging from 0 to 21 with higher scores indicating more severe distress. Items enquire about symptoms over the preceding week and are self- or clinician-rated on a 4-point Likert scale. The developers suggested categorising subjects according to subscale score into noncases (0 to 7), possible cases (8 to 10), and probable cases (>10) of clinical depression [4].

A 1997 review found both subscales to be reliable and valid measures for assessing anxiety and depression symptoms in medical patients in European, American, and Asian cohorts [6]. An updated analysis in 2002 found similar results in general medical, psychosomatic, and psychiatric patients with an optimal cut-off score of ≥8 for both subscales to define patients with probable diagnosis of depression or anxiety [7]. However, classifying patients as either depressed/anxious or not according to HADS threshold scores is controversial, especially so in chronic disease. A range of cut-offs has been used, for example, HADS-D >4 in coronary heart disease [8], HADS-D >7 in cancer [9], and HADS-D >11 in end-stage renal disease [10]. Its original purpose as a screening tool notwithstanding, in these studies, categorisation according to HADS scores is often implicitly used to diagnose depression.

The HADS has frequently been used in patients with COPD, among other reasons to assess psychological health status [11], quality of life [12], and effectiveness of pulmonary rehabilitation [13]. In spite of its widespread use, the HADS' diagnostic accuracy in COPD patients has only been examined in a small sample for anxiety [14]: in 55 COPD patients, of whom 14 were clinically diagnosed with an anxiety disorder, the optimal HADS-A cut-off score of ≥4 achieved moderate diagnostic power. However, no validation of the HADS for diagnosing depression in COPD patients has yet been attempted although the aforementioned results indicate that optimal cut-off scores for chronic disease patients are likely to differ from those originally suggested for the general patient population.

The aim of the current cross-sectional study was to validate the use of the HADS in screening patients with COPD for the presence of clinically diagnosed depression. We furthermore explored the role of patient and disease-specific predictors for depression. Data were extracted from the baseline assessment of an ongoing longitudinal COPD cohort study in Switzerland.

## 2. Patients and Methods

### 2.1. Study Subjects

Inclusion criteria were an objective diagnosis of COPD according to GOLD guidelines [15] and age between 40 and 75 years. Exclusion criteria were mental or physical disability precluding informed consent or protocol compliance, as well as acute or recent (within the preceding six weeks) exacerbation of COPD.

### 2.2. Study Design

The Obstructive Pulmonary Disease Outcomes Cohort of Switzerland (TOP DOCS) is an ongoing prospective observational cohort study coordinated by the University Hospital of Zurich, Switzerland, involving patients with mild to very severe (GOLD stage I to IV) COPD examined annually for at least three years each. Recruitment involves seven hospitals in Switzerland. A range of demographic, COPD-specific, physiologic, and quality of life-related variables are recorded. At baseline patients attending participating clinics consented and were enrolled in a nonselective, consecutive manner. The study was approved by the Ethics Committee of the Canton of Zurich, Switzerland (*Kantonale Ethikkommission Zürich*), registration reference KEK-ZH-Nr. 2011-0106.

### 2.3. Measurements

Patients' characteristics and clinical information were ascertained through self-report questionnaires, investigator-led interviews, and clinical records. Apart from details extracted from detailed clinical records, all assessments were conducted by either trained pulmonologists or dedicated study investigators.

Information about the presence or absence of an active diagnosis of unipolar depression according to ICD-10 [16] was extracted from patients' clinical records and double-checked by personal communication with patients' physicians. Most diagnoses had been made by primary care physicians (who are required to apply ICD-10 coding criteria to receive reimbursement from patients' health insurance providers). Whilst this precluded the uniform use of rigorous psychiatric interviews, the approach is an adequate reflection of clinical reality.

The German language version of the HADS [4] was administered as a self-rated questionnaire for patients to fill in either during the recruitment visit or as soon as possible thereafter. Both the HADS-A and HADS-D 7-item components were administered (each ranging from 0 to 21 with higher scores indicating increased symptoms) to evaluate patients' perceived psychological distress.

Forced expiratory volume in one second (FEV1) and maximum forced vital capacity (FVC) were assessed according to the criteria for reproducibility of the American Thoracic Society [17]. COPD-specific assessments included the 6-minute walking test (6-MWT; maximum distance in meters walked in six minutes) [18], Medical Research Council (MRC) Dyspnoea score (ranging in ascending severity from 0 to 4) [19], COPD assessment test (CAT; an 8-item health-related quality of life questionnaire ranging from 0 to 40 with higher scores indicating more severe impairment) [20], and BODE index (the composite of body mass index (BMI, body weight in kilograms divided by body height in meters squared), FEV1% predicted, 6-MWT, and MRC Dyspnoea scale; ranging from 0/low risk to 10/high risk) [21].

### 2.4. Analysis

Receiver operating characteristic (ROC) curves [22] and area under the curve (AUC) statistics were compared for HADS-D and HADS-total [23]. DeLong et al.'s [24] approach for estimating ROC parameters, Hilgers' [25] nonparametric 95% confidence interval (CI) estimation method for criterion values, and Youden's [26] index *J* (the maximum vertical distance between the diagonal guessing line and the ROC curve) were estimated alongside likelihood ratios (LR). A clinical diagnosis of depression at the time of assessment was the reference standard. Multivariable logistic regression models for depression with stepwise predictor selection were constructed including HADS, age, gender, BMI, FEV1%, number of comorbidities, 6-MWT, and MRC-dyspnoea (chosen as established risk factors for depression in COPD [3, 27, 28]). CAT score was added to control for nonspecific COPD-related quality of life impairment and antidepressant use was included to adjust for treatment-related confounding. Model assumptions were tested via Kolmogorov-Smirnov tests and residual plots. Independent sample *t*-tests, Mann-Whitney *U* tests, and Chi-Square statistics were Bonferroni-corrected for multiple testing (nominal *P* < 0.05). Analyses were performed using MedCalc for Windows, version 12.6.1 (MedCalc Software, Ostend, Belgium).

## 3. Results

**Figure 1:**
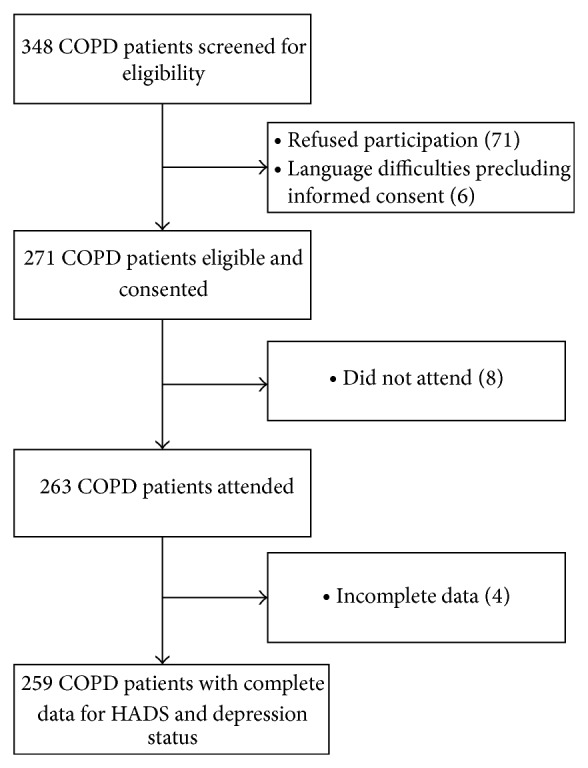
Flowchart of chronic obstructive pulmonary disease (COPD) patients in the study.

Figure 1 depicts the flow of all 263 participants enrolled between October 2009 and June 2013 from screening to analysis. Four patients were excluded due to missing data, leaving 259 COPD patients who provided complete information for HADS score and depression status. The prevalence of active depression according to ICD-10 was 11.2% (29/259). Among depressed patients, 35% (10/29) had been prescribed an antidepressant. Patient characteristics are displayed in Table 1. There were no significant differences between depressed and nondepressed patients with respect to demographic and disease-related characteristics although there was a trend for worse airflow limitation and more severe GOLD stage in nondepressed patients.

Patients with a preexisting diagnosis of depression had significantly higher HADS-D scores than nondepressed patients: median ± quartiles 6 (4; 9) for depressed versus 4 (2; 7) for nondepressed subjects (*P* = 0.004). The same held true for the HADS-total score: median ± quartiles 14 (10; 20) for depressed versus 8 (5; 14) for nondepressed patients (*P* = 0.002).

**Figure 2:**
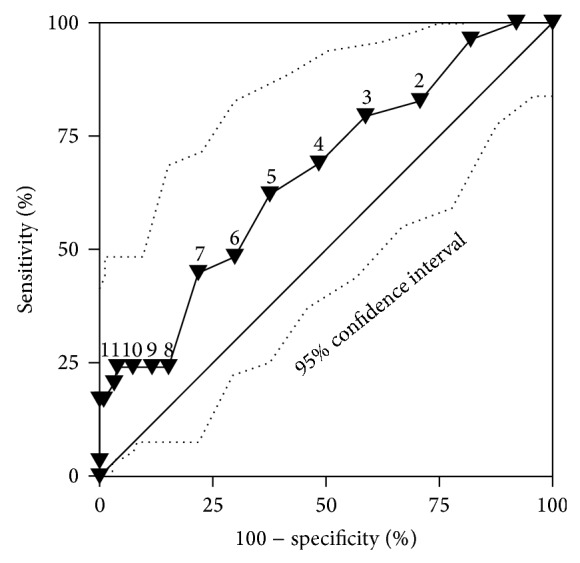
Receiver operating characteristic (ROC) curve for the HADS-depression subscale. Solid triangles indicate different cut-off scores for diagnosing depression.

**Figure 3:**
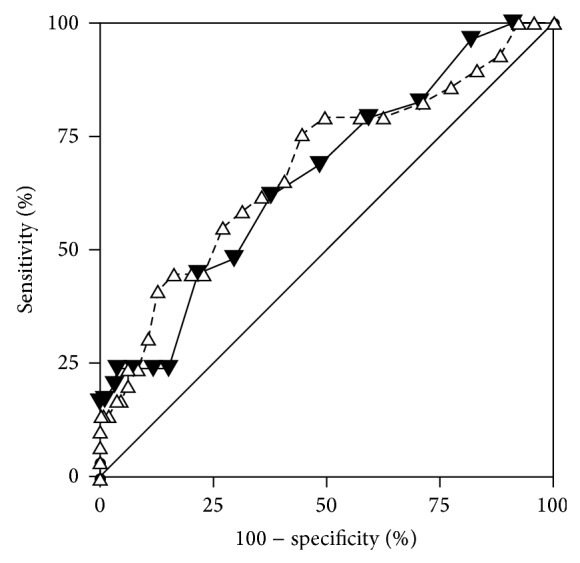
Comparative receiver operating characteristic (ROC) curves for HADS-depression (solid triangles) and HADS-total (white triangles).

Figure 2 depicts the ROC curve for the HADS-D subscale. The overall discriminant performance was low but significantly different from random chance, AUC 0.66 (95% CI 0.60–0.72). Youden's index *J* identified a threshold of HADS-D >5 as the optimal cut-off score to diagnose depression (*J* = 0.25). A cut-off >5 yielded a sensitivity of 62.1% (95% CI 42.3%–79.3%), a specificity of 62.6% (95% CI 56.0%–68.9%), a positive LR of 1.66 (95% CI 1.2–2.3), and a negative LR of 0.61 (95% CI 0.4–1.0). Using a cut-off value of 5 on the HADS-D applied on the background of an observed prevalence for depression of 11.2% yielded a positive predictive value of 17.3% and a negative predictive value of 92.9%. Thus, in 100 hypothetical patients with a HADS-D >5, 17 will qualify for a diagnosis of depression, whilst among 100 patients with a score ≤5, on average 93 will be correctly identified as nondepressed. The comparison between HADS-D and HADS-total ROC plots is shown in Figure 3. There was no significant difference in overall performance between both scores, AUC_HADS-D_ 0.662 versus AUC_HADS-total_ 0.681 (95% CI for the difference 0–0.070). The optimal cut-off score for diagnosing depression on the HADS-total was >9, Youden's *J* = 0.311, sensitivity 75.9% (95% CI 56.5%–89.7%), and specificity 55.2% (95% CI 48.5%–61.8%).

The stepwise logistic regression model identified four significant predictors (model fit *P* < 0.001). An increasing likelihood of suffering from depression was predicted by lower age (*P* = 0.010), higher number of comorbidities (*P* = 0.004), male gender (*P* = 0.016), and higher percentage of predicted FEV1 (*P* = 0.047). This model correctly classified 89.6% of cases. Controlling for antidepressant medication use did not alter the results. Thus, younger men with additional comorbidities had the highest risk of depression.

## 4. Discussion

In this cross-sectional multicentre cohort study involving patients with mild to very severe COPD, we found low accuracy of both the HADS-D and HADS-total in identifying patients with a preexisting diagnosis of depression. As the optimal cut-off HADS-D >5 yielded a positive predictive value of only 17.3% and a negative predictive value of 92.9%, the test seems to more accurately identify the absence rather than presence of depression. Its usefulness as a general measure of psychological distress notwithstanding, the validity of the HADS-D as a tool for classifying COPD patients into depressed and nondepressed categories—as commonly applied in previous studies—is questionable. This lack of discriminant power may be explained in part by the original validation of the questionnaire, which was aimed at a general medical case mix in an outpatient setting, rather than at secondary/tertiary care patients with chronic debilitating ailments. Yet, despite caveats mentioned by the HADS' developers [5], over the last three decades it has been applied to the evaluation of depression and anxiety symptoms in a large variety of clinical contexts outwith its original target group. Relying on a one-week retrospective questionnaire-based approach to classify patients as depressed or not for ensuing subgroup analyses (e.g., to predict physical activity [29]) is not appropriate. Moreover, the original purpose of the HADS as a screening rather than diagnostic tool should forbid reliance on its results as the sole indicator of clinically significant depression—a labelling approach applied in previous research. This study reemphasizes that the HADS should not be used to diagnose depression or reliably subgroup patient samples.

Our results on depression in COPD patients are in line with findings from a recent meta-analysis in cancer and palliative care patients [30], which reported a weighted combined sensitivity of 71.6% with a specificity of 82.6% of the HADS for identifying depression. Restricting analyses to trials using HADS-D >7 as cut-off yielded a sensitivity of 68.3% with a specificity of 85.7%. The authors of the meta-analysis promote the use of the HADS as a screening rather than diagnostic tool. High subscale correlations may favour using the HADS as a general measure of psychological distress rather than specifically detecting depression and anxiety [31–33]. Furthermore, studies vary substantially in cut-offs used to identify mental morbidity, casting doubt on the HADS' usefulness as a screening tool [34]. Using recommended cut-off scores may underestimate psychiatric morbidity in cancer patients [35].

A review incorporating all studies (2000 to 2010) investigating the HADS' factor structure found heterogeneous results: only half of the included trials confirmed the two-factor model (depression and anxiety), whilst others identified between one and four underlying constructs [31]. For example, an established alternative model suggests three factors labelled, respectively, “negative affectivity,” “anhedonic depression,” and “autonomic anxiety” [32]. Yet, a 2013 metaconfirmatory factor analysis favoured the depression/anxiety two-dimensional structure [33]. Given the confusing theory underpinning the HADS, its use as a measure of any specific psychiatric disorder should best be avoided.

Consequently, the inconsistency of the HADS' factor structure across samples [31], the discrepancy between its wordings based on colloquial British expressions, and its international application [36], compounded by the exclusion of somatic items, have led to calls for abandoning the 30-year-old HADS in favour of more accurate instruments [37]. Others continue to promote the HADS as a valid, cross-culturally appropriate tool for assessing psychological distress [38]. Future studies should make an effort to validate psychiatric diagnoses in line with the best clinical practice.

The prevalence of depression according to ICD-10 in our cohort (11.2%) was lower than in other studies with reported rates of up to 42% [27]. However, considering only studies based on a definition of depression according to established classification systems yields a lower estimate of about 20% [39]. Another explanation for the low prevalence of depression may be the homogeneous composition of our cohort composed of well looked-after patients recruited from established high-quality care centres in Switzerland. The observed higher rate of diagnosed depression in younger patients with better airway function could be due to a lower threshold for seeking professional help for psychological issues in more recent generations. Less physically limited patients may be more inclined to recognising and addressing ailments other than pulmonary disease. Alternatively, clinicians' thresholds for diagnosing depression could be different in these patients. Many factors may contribute to emotional morbidity in COPD patients, including social isolation and dependence on others for activities of daily living [28]. Crucially, however, feelings of low mood and the general psychological impact of chronic disease must not be equated with a psychiatric diagnosis of a depressive disorder. The HADS gauges psychological impairment but is not suited as a diagnostic tool. Investigators need to be cautious about labelling subjects inappropriately. As confirmed by our results, overreliance on a self-report questionnaire is prone to erroneous categorisation of patients.

There are some limitations to our study. The recruitment context of specialist pulmonary care necessitated the evaluation of preexisting rather than newly diagnosed depression. Whilst recorded diagnoses of depression were double-checked with patients' registered clinicians, short-term variations in mood may have hampered the HADS' ability to detect long-term impairment. Yet, as many clinical trials have used the HADS to label patients as depressed or not irrespective of any reference standard, our findings carry significant implications for the planning of future trials. The comparatively low prevalence of depression, potentially contributed to by underdiagnosis, may have limited the statistical power. Nonetheless, our large nonselective sample is representative of the COPD patient population in Switzerland and is consequently characterised by a high socioeconomic status and low proportion of ethnic minorities—both factors that have been linked to low rates of depression [40].

## 5. Conclusions

In this large cross-sectional study of stable COPD patients, the HADS questionnaire had a low accuracy in identifying a diagnosis of depression. This is the first study to address HADS-D test accuracy in COPD patients. Depression rates were highest among young male patients with additional comorbidities. The clinical implications of our findings are twofold. Firstly, the HADS should not be used as a stand-alone diagnostic tool for depression in COPD patients. It provides an appropriate scale to evaluate psychological distress but does not allow for diagnostic classification. Secondly, clinicians' awareness of the significant prevalence of psychological comorbidities in chronic pulmonary disease patients needs to be improved.

## Figures and Tables

**Table 1:** Sample characteristics.

Variable	Not depressed (*n* = 230)	Depressed (*n* = 29)	*P* value^†^
Female, *n* (%)	70 (30.4%)	14 (48.3%)	0.085
Age, mean (SD)	62.7 (7.5)	60.0 (8.3)	0.057
BMI, mean (SD)	26.4 (6.1)	26.1 (5.9)	0.808
Comorbidities, mean (SD)	2.4 (2.2)	3.1 (1.6)	0.061
Diabetes mellitus, *n* (%)	32 (13.9%)	1 (3.4%)	0.195
OSA, *n* (%)	21 (9.1%)	5 (17.2%)	0.298
Arterial hypertension, *n* (%)	109 (47.4%)	15 (51.7%)	0.808
Malignancy, *n* (%)	27 (11.7%)	3 (10.3%)	0.931
GOLD stage, *n* (%)^††^
I	17 (7.4%)	2 (6.9%)	0.035
II	74 (32.2%)	16 (55.2%)	
III	87 (37.8%)	10 (34.5%)	
IV	52 (22.6%)	1 (3.5%)	
CAT, mean (SD)	15.7 (7.1)	17.4 (7.4)	0.213
BODE index, mean (SD)	3.4 (2.4)	2.8 (2.3)	0.203
MRC-dyspnoea, mean (SD)	1.7 (1.1)	1.8 (1.0)	0.736
FEV1%, mean (SD)	46.6 (20.7)	54.5 (21.2)	0.044
6-MWT (meters), mean (SD)	412 (131)	410 (124)	0.916

^†^Frequencies and means were compared using *χ* ^2^-tests and independent *t*-tests, respectively.

^††^ *χ* ^2^-test for the frequency distribution across GOLD stages I/II/III/IV in depressed versus nondepressed patients.

6-MWT: 6-minute walking test; BMI: body mass index; CAT: chronic obstructive pulmonary disease assessment test; CPAP: continuous positive airway pressure; FEV1%: percentage of predicted forced expiratory volume in one second; GOLD: global initiative for chronic obstructive pulmonary disease; MRC-dyspnoea: Medical Research Council dyspnoea scale; OSA: obstructive sleep apnoea.
